# Planar Thermoelectric Microgenerators in Application to Power RFID Tags

**DOI:** 10.3390/s24051646

**Published:** 2024-03-02

**Authors:** Andrzej Dziedzic, Szymon Wójcik, Mirosław Gierczak, Slavko Bernik, Nana Brguljan, Kathrin Reinhardt, Stefan Körner

**Affiliations:** 1 Wrocław University of Science and Technology, Wybrzeże Wyspiańskiego 27, 50-370 Wrocław, Poland; andrzej.dziedzic@pwr.edu.pl (A.D.);; 2 Jožef Stefan Institute, Jamova cesta 39, 1000 Ljubljana, Slovenia; 3 Fraunhofer IKTS, Winterbergstraße 28, 01277 Dresden, Germanystefan.koerner@ikts.fraunhofer.de (S.K.)

**Keywords:** energy harvesting, thermoelectricity, thick film, thermocouple, thermopile, thermoelectric microgenerator, RFID tag

## Abstract

This paper presents an innovative approach to the integration of thermoelectric microgenerators (μTEGs) based on thick-film thermopiles of planar constantan–silver (CuNi-Ag) and calcium cobaltite oxide–silver (Ca_3_Co_4_O_9_-Ag) thick-film thermopiles with radio frequency identification (RFID) technology. The goal was to consider using the TEG for an active or semi-passive RFID tag. The proposed implementation would allow the communication distance to be increased or even operated without changing batteries. This article discusses the principles of planar thermoelectric microgenerators (μTEGs), focusing on their ability to convert the temperature difference into electrical energy. The concept of integration with active or semi-passive tags is presented, as well as the results of energy efficiency tests, considering various environmental conditions. On the basis of the measurements, the parameters of thermopiles consisting of more thermocouples were simulated to provide the required voltage and power for cooperation with RFID tags. The conclusions of the research indicate promising prospects for the integration of planar thermoelectric microgenerators with RFID technology, opening the way to more sustainable and efficient monitoring and identification systems. Our work provides the theoretical basis and practical experimental data for the further development and implementation of this innovative technology.

## 1. Introduction

With the rapid development of technology and the increasing demand for modern and sustainable energy sources, scientists and engineers are paying increasing attention to innovative solutions in the field of thermoelectric microgenerators (μTEGs). An area of research is the use of μTEGs in combination with radio frequency identification (RFID) technology. This combination not only opens up new prospects in the field of autonomous energy sources but also enables the development of advanced tracking and identification systems.

Thermoelectric microgenerators (μTEGs), based on the Seebeck phenomenon, allow the conversion of temperature difference into electrical energy. Using this phenomenon creates the possibility of powering small electronic devices such as sensors, transmitters, or even RFID tags. In the context of RFID applications, μTEGs open new horizons for autonomous power and secure identification.

RFID technology, which relies on the wireless transmission of data using radio waves, is widely used in various fields such as logistics, transportation, inventory management, and sensorics. However, traditional power sources for active RFID tags, such as batteries, can generate problematic environmental pollution and require regular replacement, which in turn can lead to loss of functionality and cost increase.

Combining thermoelectric microgenerator technology with RFID opens up a number of potential applications in various industries, mainly due to the specific requirements of this technology. μTEGs operate effectively in the presence of a temperature gradient, which opens up possibilities for a number of practical applications.

One potential application area is manufacturing. It is possible to use heated components inside production machines, which can generate large temperature gradients between the hot component and the environment. Another area is the automotive industry, where many components, such as the engine or exhaust pipe, reach high temperatures during the fuel combustion process. Using a microgenerator in such a context makes it possible to heat one side of the thermocouple with hot elements while cooling the other side as a result of the air movement generated during driving.

The possibilities associated with cooling one side of the microgenerator instead of heating it are also under consideration. In the medical field, where special freezers are used to store medical preparations, it is possible to imagine a situation where one side of the microgenerator is attached to human skin and the other side is exposed to extremely low temperatures as low as −30 °C.

The space industry also represents a promising market for applications of this technology, mainly due to low temperatures in space. One side of the microgenerator can be placed outside, where it is exposed to extreme conditions, while the other side can be placed in an environment tuned for human life, such as inside a space station or rocket.

This article presents the possible use of planar μTEGs in the context of powering RFID tags (active or semi-passive). Verification of the benefits of this relationship, analysis of technological possibilities, and prospects for further development in this area will be undertaken.

The main purpose of the analysis was to explore the potential of combining active or semi-passive RFID tags with planar thermoelectric microgenerators (μTEGs) due to their characteristics. RFID tags with an additional power supply offer significantly better communication performance, such as signal strength, resulting in a longer communication range. With appropriate μTEGs, it is possible to consider situations in which the RFID tag is self-sustaining and does not require additional intervention throughout the life of the device while generating a signal strength comparable to tags powered by traditional sources such as batteries.

Due to the distinctive characteristics of μTEGs, they allow the extraction of energy from the environment, converting thermal energy into electrical energy. This ability means that the microgenerators can be used effectively only where there is a temperature gradient. This results in the fact that the implementation under consideration cannot be applied universally. The main area of application is where there is a natural temperature gradient. Instead of wasting this energy, it can be efficiently used to power active or semi-passive RFID tags. Due to the specific requirements of μTEGs, they do not compete directly with other types of energy conversion, such as solar, mechanical, or RF. They are a substitute that expands the range of power options. The final choice between thermal, solar, or mechanical energy conversion and traditional battery systems to power RFID tags depends on the environmental conditions at the site.

This paper attempts to simulate planar thermoelectric microgenerators in various configurations. Based on tests conducted for actual microgenerators, simulations were performed with structural modifications to achieve the proper thermoelectric strength and power necessary for efficient operation in combination with a potential RFID tag. The objective of the analysis was to determine whether such a combination is possible.

To achieve this, in the first stage, two material systems—Ca_3_Co_4_O_9_-Ag and CuNi-Ag—were experimentally studied and used to fabricate planar thermoelectric microgenerators in thick-film technology. Based on the measurements of the thermoelectric force generated by them and changes in their internal resistance as a function of the temperature difference between cold and hot thermoelectric junctions, this is as a function of the temperature gradient, and the parameters of individual thermocouples were determined, i.e., the characteristic dependence of their Seebeck coefficient and internal resistance (and thus resistivity) on temperature.

In the next step, the values of the thermoelectric force and internal resistance of planar μTEGs were simulated depending on the number of thermocouples in the thermopile, the geometric dimensions of the arms of a single thermocouple, the reference temperature, and the value of the temperature gradient. The obtained results were compared with the energy requirements of the RFID tags, which led to the conclusion that the proposed solutions could find applications to power active or semi-active RFID tags.

In the future, the practical implementation of this solution is planned, which, according to simulations, should work properly under the relevant conditions defined in this paper.

## 2. Thermoelectric Microgenerators (μTEGs)

μTEGs are devices that convert the temperature difference between two junctions of conductors or semiconductors into electrical energy using the Seebeck effect. This type of technology is widely used and is gaining popularity due to its features [1]. The main component of a microgenerator is the thermoelectric module, also called the thermopile, which consists of many pairs of thermocouples. The most common materials in microgenerators are usually bismuth, tellurium, and lead-based compounds [2]. These materials have thermoelectric properties that allow them to efficiently convert the temperature difference into electrical energy.

Thermoelectric structures have different parameters due to the materials used. To verify the performance of the structures, appropriate correlations are used, allowing the different parameters to depend on each other. The most common parameters are the power factor (PF), figure of merit (Z), and the dimensionless figure of merit (ZT).

(1) $$PF=\frac{\alpha^{2}}{\rho }\;\left[\frac{W}{m\times K^{2}}\right]$$

(2) $$Z=\frac{\alpha^{2}}{\rho \times \lambda }=\frac{PF}{\lambda }\;\left[\frac{1}{K}\right]$$

(3) $$ZT=\frac{\alpha^{2}}{\rho \times \lambda }\times T=\frac{PF}{\lambda }\times T=Z\times T\left[-\right]$$

where α is the Seebeck coefficient, ρ is the resistivity, λ is the thermal conductivity, and T is the temperature.

We can distinguish two main modes of operation of microgenerators, constant and variable. In the first case, there is a constant temperature gradient between junctions, and then the microgenerator generates a constant value of thermoelectric force and thus a constant electrical power. In the variable mode, on the other hand, the TEG generates a variable value of electrical power due to random or periodic changes in the temperature gradient. This is because the ambient temperature may not be constant and thus the voltage generated by the structure is variable. When selecting a microgenerator, it is necessary to take into account the minimum and maximum temperatures between the junctions and make sure that it will be sufficiently effective in extreme cases. Otherwise, the microgenerator may provide less energy than expected, and, as a result, the system will not work properly without another power source.

μTEGs have found applications in powering low-voltage devices such as sensors, clocks, and small electronic portable devices [3]. They are used in health monitoring systems, where they can power various actuator components [4]. A receiving group is also the automotive industry, where thermoelectric microgenerators can convert the heat generated during the operation of an engine or other components into additional electrical energy [3]. In the industry, μTEGs can be used to generate electricity where there are temperature differences between production processes.

Despite their many advantages, μTEGs face challenges, such as improving conversion efficiency, reducing manufacturing costs, and developing more efficient thermoelectric materials. However, with continued advances in science and technology, μTEGs have the potential to become an important component in the field of sustainable energy and portable electronics [5].

## 3. RFID Tags

RFID technology allows for contactless identification of objects using radio waves. An RFID system consists of two main components: a reader (or readers) and RFID tags [6,7]. The RFID reader and the RFID tag are the two key components of RFID-based identification systems. These two elements work together to create an efficient way to track, identify, and manage data. However, there is a fundamental difference between them, both in terms of function and the role they play in the system.

An RFID reader is a device that has the ability to receive and interpret radio signals transmitted by RFID tags. It acts as the interface between the tag and the data management system. The reader is usually a powered device that actively sends radio signals toward the tags and receives responses while identifying each tag’s unique identification number. RFID readers are widely used in various fields, such as logistics, warehousing, transportation, and access control systems [6,7].

An RFID tag is a small and usually inexpensive device that contains an antenna and a microchip or other form of memory. It stores information, such as an identification number or data about the product or object to which the tag is attached. RFID tags often do not have their own power source as they are activated by energy from radio signals sent by the RFID reader when approached—these are passive tags. There are also active tags with their own power supply or semi-passive tags that combine the functionality of active and passive tags. There are many different types of RFID tags, including those designed for one-time use and more advanced reusable ones [6,7].

Passive tags are often cheaper, smaller, and lighter, making them ideal for applications where size constraints are important. Passive tags can be used in areas such as product marking, inventory management, logistics, or access control. They can also work with various types of sensors, but a power supply is often required. Active RFID tags, unlike passive tags, have their own power source (usually a battery). As a result, they can transmit data over longer distances and offer additional functions. They are usually larger and more expensive. They are used, for example, in vehicle tracking, real-time monitoring, or access control systems. Semi-passive tags have their own power source but also use energy from the reader during communication. They represent a compromise between the range of active tags and the smaller size of passive tags. Their application area is a combination of applications for active and passive tags [8,9,10,11].

Communication between the reader and the tag can follow two basic communication procedures called full/half duplex (FDX/HDX) or sequential (SEQ). Both communication methods can be used for all types of tags. In multidirectional mode (full duplex mode), the tag and the reader can transmit data simultaneously in both directions. This means full two-way communication. To support this mode, a continuous power supply to the tag is required for the period of communication. In the single-directional mode (half duplex), communication between the tag and the reader is alternate. Only one device can transmit data at a time. A continuous power supply to the tag for the period of communication is required to support this mode. In sequential mode, communication between the tag and the reader is alternated. At any given moment in time, only one device transmits information. The sequential mode is characterized by the fact that only a temporary power supply to the tag is required [12].

With different types of RFID tags and modes of operation, the technology is used in a wide range of fields, from logistics to security systems, bringing significant improvements in the identification, tracking, and management of various types of objects.

## 4. Powering RFID Tags—A Short Literature Review

The planned integration of μTEG with RFID tags requires the determination of the electrical parameters necessary for the proper operation of the system. Based on the literature, it can be observed that the minimum voltage needed to power the microprocessor is in the range from 1 V to 1.5 V [13,14,15,16]. An RFID chip powered by 1.5 V, which is mainly due to the processor used is described in [13]. Due to the widespread use of 1.5 V batteries, many low-voltage systems are based on this voltage. This trend is also described in [15], where a special processor has been designed for 1.5 V so as to achieve low current consumption. The main reason why they operate at voltages above 1 V is the processor [13,16]. At this very moment, circuits operating at low voltages are being researched, which is also shown in [16], where a special processor for RFID tags that can operate from 1 to 1.8 V is presented. For example, it is shown that the RFID tag works correctly with a power supply voltage of 1.2 V [14].

However, it should be noted that the basic parameter that defines the operation of the system is the power. The minimum power defined in the literature for the basic operation of an RFID tag is 8 μW. Such a low power is already sufficient to read basic information such as ID [14]. With specially designed systems this value can be even lower; the literature reports that in extreme conditions it is even possible to operate at a power of 3.15 μW [15]. However, it is generally assumed that the commonly accepted range of power is from 8 μW to 1 mW [7,14]. Note that when calculating power, it must be taken into account that the power generated by the energy source is not the final power. Assuming resistance matching of the generator and the load, ¼ of the generator power should be assumed [13].

Due to the application, various sources of renewable energy are used to power RFID tags. The basic principle for all types of conversion is to acquire ambient energy and then, using appropriate conversion circuits, accumulate it together with RF energy. This solution allows for proper functioning, ensuring continuous operation. Applications are described, for example, in [17], which focuses on an overview of solutions. Photovoltaic conversion is suitable for general applications, especially for supply chains, construction sites, and industry. RF conversion is also suitable for general applications, medicine, and agriculture. In addition, the area of interest is thermal conversion (using TEG), which can be excellent for industrial and medical care [17]. Of course, application areas can be very broad for all methods. The most important is the general scheme for connecting an RFID tag with a harvester chip. Due to the processors used, it is very important to provide a constant and relatively high voltage. For this purpose, suitable step-up converters should be used, which provide the required electrical parameters to power the processor chip and the RFID tag interface.

Very often, unconventional energy sources are proposed to power RFID tags. This is connected with problems during the use of RFID tag sensors, which relied mainly on batteries. For example, an RFID system powered by a small, flexible photovoltaic panel is described in [18]. During real-world measurements, the indoor reading range was up to 23 m. The entire system required about 168.3 µW. Of course, the processed energy must meet minimum power criteria. The energy collected thus is then stored using, for example, capacitors or batteries [10]. In the end, the harvested energy can be a component for powering the RFID tag.

Piezoelectric energy conversion is also commonly considered. A suitable generator can convert the shock energy of various naturally occurring mechanical vibrations into electrical energy. In [19], the authors attempted to analyze the combination of RFID tags and a piezoelectric generator due to the need for periodic battery replacement. The authors elaborate on possible applications of piezoelectric generators in active RFID tags. As examples of applications of RFID tags with piezoelectric generators, they proposed, for example, a security system to monitor children going to and returning from school and a network system to monitor the use of a conference room [19]. A microvibrating MEMS system may also find its application [20]. The energy provided by the vibrating system is stored in a capacitor and released by a mechanical switch to a tag. During tests, communication of up to 15 m was possible when the chip was powered by a microvibrator [20].

The literature also describes combinations of thermoelectric technology with RFID tags. The integration of an RFID tag with a flexible thermoelectric generator (TEG) is presented in [21]. It consists of an EPC C1G2/ISO 18000-6C ultrahigh-frequency RFID integrated circuit connected to a low-power microcontroller unit [22]. With a gradient of 2.5 °C, the TEG generates an output power of 400 μW at an output voltage of 40 mV. Through the use of a step-up converter, the appropriate electrical values are supplied to the RFID tag. During semi-passive operation, a communication range of up to 22.2 m has been measured [21]. It is also possible to combine the antennas with a thermoelectric generator [23]. The TEG can be mounted on top of the antenna, and its main task is to convert a thermal gradient between the antenna and the environment. The antenna is properly designed to ensure good heat transfer between the antenna and the TEG [23].

An interesting application is the design of a battery-free wearable system that measures the skin temperature of the human body while collecting energy from body heat using TEG. The system consists of a UHF RFID temperature sensor placed on the patient’s arm, supported by additional power from an energy harvesting module that collects the thermal energy emitted by the patient’s body. The experimental results of thermal energy harvesting are presented, and the module is characterized under various conditions, such as immobility and indoor and outdoor walking [24]. Finally, the tag was tested under fully passive and assisted operating conditions. The results show that the communication range of the RFID sensor is improved by 100% when taking measurements every 750 ms and by 75% when taking measurements every 1000 ms when the sensor is assisted by the energy harvesting module.

A hybrid system that combines radio frequency (RF) signals and energy harvested thanks to thermoelectric phenomena was also proposed. The main objective was to combine energy from both sources to solve the limited lifetime of semiactive UHF RFID tags, which are battery-powered due to the specific requirements of their applications. The simulation results show that the system can operate even at very low input voltages, such as 31.62 mV, and is capable of harvesting energy from a single source or both sources simultaneously. From the simulations, the maximum output voltage of 3.44 V was obtained from a single RF source of 0 dBm. A maximum output voltage of 3.40 V was obtained when energy was collected from both sources, RF and thermoelectric, simultaneously, with a power of 0 dBm and a temperature difference of 10 K [25].

On the basis of the literature, we see that TEG technology is being considered in combination with RFID tags. Our most important goal is to verify whether specific materials, Ca_3_Co_4_O_9_ and CuNi, have the potential to be used as energy sources for RFID tags. We plan to use a standard step-up converter to increase the voltage to the required level. In contrast to other publications, our application is based on a planar thermoelectric microgenerator (μTEG) instead of a generator (TEG). This results in a much smaller size (volume), which helps to ensure the compactness of the device. It is worth noting that due to the very small dimensions of commonly available RFID tags, microgenerators allow the final device to maintain very small dimensions. The general trend toward miniaturization also works in favor of the presented solution, and the simulations themselves are preliminary for further research.

## 5. Planar Thermoelectric Microgenerators (μTEGs)—Own Research

**Figure 1:**
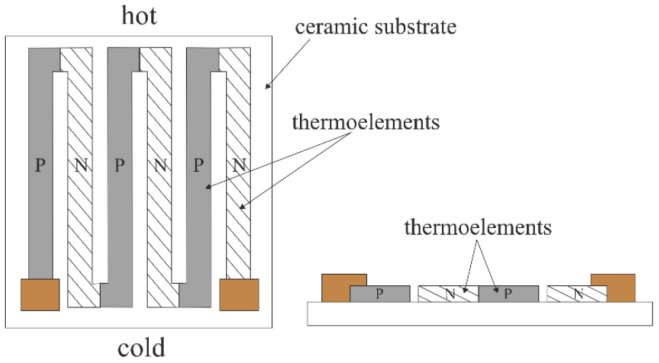
Schematic of a planar thermoelectric microgenerator (μTEGs).

**Figure 2:**
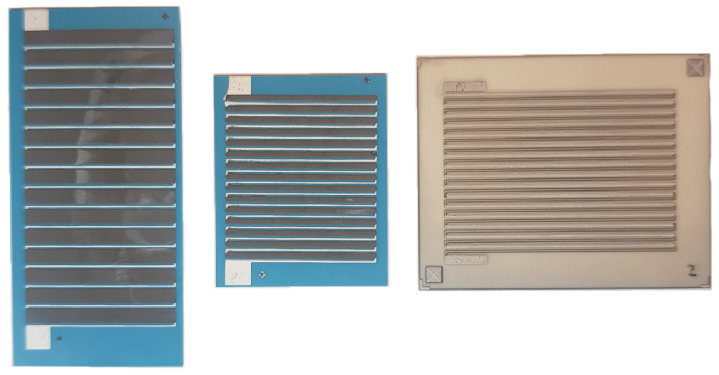
Examples of planar μTEGs.

The research conducted was carried out using planar μTEGs (Figure 1 and Figure 2). A key feature of planar TEGs is their flat structure, which means that they are built on a single plane. The flat structure of μTEGs has important applications in microsystems where small size is key. Due to their compact design, the systems are finding applications in fields such as microscale electronics, sensorics, medicine, or other areas where there is a need for an efficient, small, and self-sustaining energy source. Planar structures make it possible to power microelectronics in places that are hard to reach or provide autonomous energy for microsystems, eliminating the need for an external power source. Planar thermoelectric microgenerators thus represent a step forward in the development of microelectronics technology, opening up new perspectives for applications that require small and self-sustaining power sources. Planar structures consist of relatively thin thermoelectric layers placed on a flat substrate. The material is spread in a single plane, causing heat to flow along the substrate [5,26,27,28,29].

A resistive paste, whose main inorganic ingredient was constantan powder (Cu_55_Ni_44_Mn) [30], a paste prepared from powdered calcium cobaltite oxide (Ca_3_Co_4_O_9_–Ca349) [31], and a commercial silver-based paste ESL 599E were used to fabricate thermopiles, which were composed of four thermocouples connected in series.

**Figure 3:**
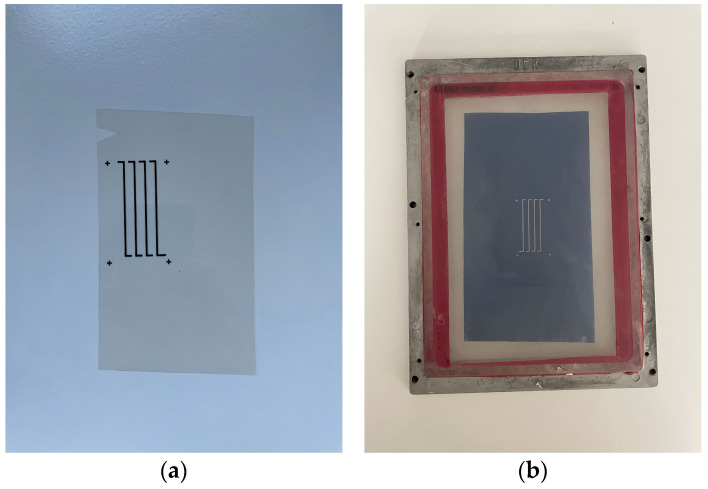
(**a**) Photolithography mask; (**b**) screen printing screen.

The test structures were realized using classic thick-film technology. For this purpose, photolithographic masks were made in the first step, and then screens were made; the mask and screen for one material are shown in Figure 3. Stainless screens with a density of 200 mesh were used. The next step was the screen printing of pastes on alumina substrates, for which a semi-automatic Uniprint PMGo3V screen printer was used. After applying, the first arms (pastes with the functional phase CuNi or Ca_3_Co_4_O_9_ functional phase) were applied and the layers were dried at 120 °C for 10 min, after which a part of the structures was subjected to isostatic compression at 200 bar. Films prepared this way were fired in a six-zone tunnel furnace BTU QA-41-6-54 on a 60 min cycle with a 10 min hold at peak temperature. The second arms (from Ag-based ink) were then printed and, after drying, fired at 500 °C peak temperature. The CuNi-Ag structures were fired in a nitrogen atmosphere, while the Ca_3_Co_4_O_9_-Ag thermopiles were fired in an air atmosphere. The basic technological steps are listed in Table 1.

**Figure 4:**
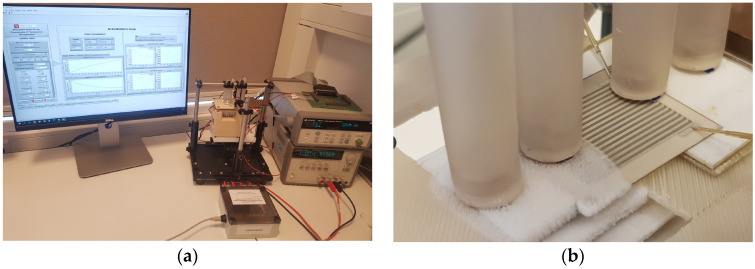
(**a**) Measurement stations for the characterization of thermoelectric microgenerators; (**b**) example of a characterization process.

The parameters of the CuNi-Ag and Ca_3_Co_4_O_9_-Ag microgenerators were determined over a wide temperature range. Measurements were carried out on a specially prepared measurement stand, the purpose of which was to properly heat/cool the structure and to perform point measurements of temperature, thermoelectric force, and internal resistance of microgenerators [32]. The stand is based on the cooperation of three modules: a desktop computer with a suitable application, a data logger, and a measuring table with a controller. After selecting the appropriate mode of operation, communication with the data logger and the controller is initiated. This enables correct operation and continuous data collection during measurements. Figure 4a shows the measuring station. Meanwhile, the arrangement of the measurement probes in the characterization of a planar thermoelectric microgenerator on an alumina substrate can be seen in Figure 4b.

Measurements of the thermoelectric properties of the test structures over a wide temperature range were carried out in two stages. In the first stage, one side of the μTEG structure is maintained at room temperature, while the other side is gradually heated to about 450 K using a thick film heater. In each successive step of 10 min, the heater power was increased by 10%. After the structure was cooled to room temperature, a stepwise cooling of the other side was performed to about 230 K using a Peltier module. In this stage, each step took approximately 7 min to stabilize the temperature on the cooled side of the structure. To determine the thermoelectric properties of the structures, the thermoelectric force and internal resistance values of the μTEGs measured under quasi-static conditions, that is, at the end of each heating or cooling step, were used.

**Figure 5:**
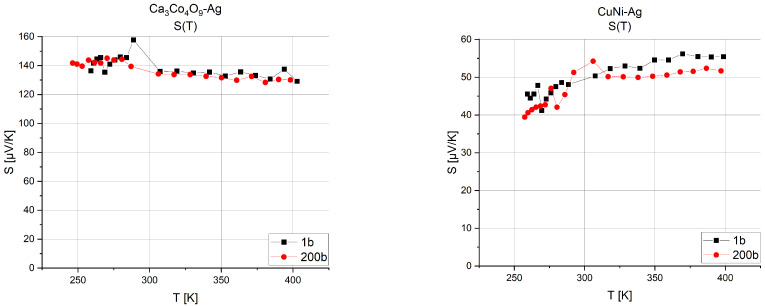
Seebeck coefficient versus temperature for compressed and uncompressed Ca_3_Co_4_O_9_-Ag and CuNi-Ag structures.

Research made it possible to determine the Seebeck coefficient and the resistivity of the structure. From the measurements, the point Seebeck coefficient (small increment defined in Equation (4)) was determined, and the parameters were converted for a single thermocouple. The graphs (Figure 5) show the change in the Seebeck coefficient versus operating temperature for two different materials. The graphs presented show that the Seebeck coefficient is not a constant parameter, and that the operating temperature matters for the final performance of the thermoelectric microgenerator. Determining the Seebeck coefficient for a single thermocouple makes it possible to define how many thermocouples to apply to obtain the desired supply voltages.

(4) $$S\left(T_{n}\right)=\frac{E\left(T_{n}\right)-E\left(T_{n-1}\right)}{T_{n}-T_{n-1}}$$

where: $T_{n}\approx T_{n-1}+10\;K$.

**Figure 6:**
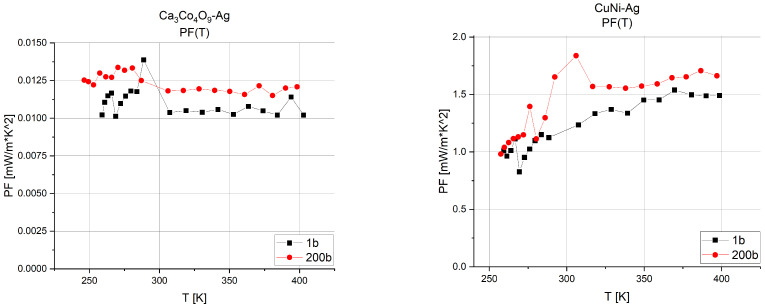
Power factor versus temperature for compressed and uncompressed Ca_3_Co_4_O_9_-Ag and CuNi-Ag structures.

On the basis of the value of the Seebeck coefficient and resistivity, the power factor (PF) was determined (Figure 6), which allows the determination of the power output of a single thermocouple. It allows the selection of a material with the best power properties at a given operating temperature. This is a very important parameter that includes the value of the Seebeck coefficient and the resistivity of the material.

## 6. Combining μTEGs with RFID

Based on the literature, it can be observed that active RFID tags require a minimum voltage of 1 V to 1.5 V for proper operation [13,14,15,16] and a power level of 8 μW to 1 mW [7,13,14], depending on the type of tag. When appropriate measures are taken for the generation of a microgenerator, the resulting parameters will allow the system to work properly. As a result, the device will be able to communicate over a longer distance and will not rely only on the energy captured from radio waves. It is also important that the tag can operate in semi-passive mode, which is even the preferred method of cooperation between RFID tags and thermoelectric microgenerators. This significantly reduces power requirements, and the main task is to increase the communication distance [21,23,24,25]. Based on the results obtained for thermoelectric structures, CuNi- and Ca_3_Co_4_O_9_-based, it can be concluded that a suitable combination of microgenerators together with RFID tags is possible. The resulting device can act as a semi-passive or active tag.

According to the measurement method described in Section 5, the heating ranges were selected for the Ca_3_Co_4_O_9_-Ag- and CuNi-Ag-based structures (structures without isostatic compression), and then the corresponding simulations were performed to determine the parameters of the thermoelectric force and the power factor depending on the number of thermocouples and the resistance of the system.

**Figure 7:**
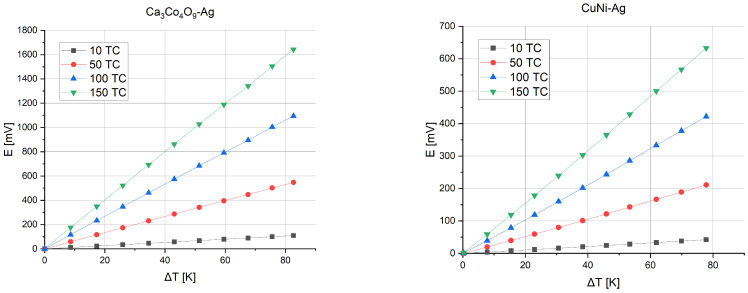
Thermoelectric force versus temperature gradient and number of thermocouples.

The first possibility is to increase the number of thermocouples on the substrate. Figure 7 shows the voltage that can be obtained depending on the temperature gradient between the junctions for microgenerators consisting of 10, 50, 100, or 150 thermocouples.

To calculate the power, it is necessary to take into account the change in resistance that occurs with a change in temperature. The resistance for a single thermocouple is presented in Table 2.

According to Ohm’s law, on the basis of the resulting voltage and resistance, it is possible to determine the power generated by the microgenerator.

(5) $$P=\frac{U^{2}}{R}$$

It is important to note that, for example, in the case of a DC circuit when matching due to the maximum power transferred from the generator to the load, which occurs when the resistance of the load is equal to the internal resistance of the source, the power given to the load is equal to ¼ of the power generated by the thermoelectric microgenerator.

**Figure 8:**
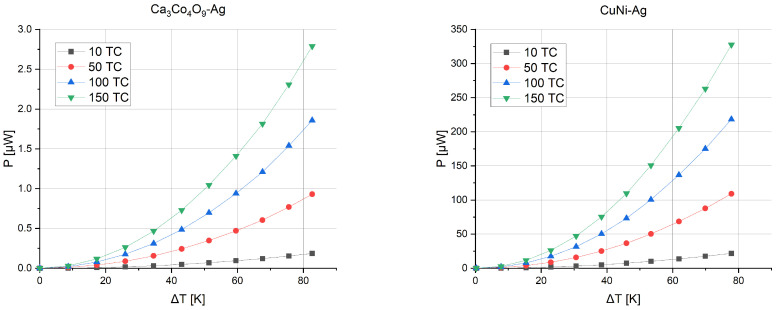
Generated power versus temperature gradient and number of thermocouples.

Figure 8 presents the power obtained from such structures. As the temperature gradient increases, the voltage and, as a result, the power that the structure is able to generate also increase.

Another possibility is to reduce the resistance of a single thermocouple. This will not affect the generated thermoelectric force because the Seebeck coefficient is a material parameter. But, on the other hand, it will allow more current to flow. When the internal resistance of a single thermocouple is reduced, for example, by 50%, respectively, it is possible to obtain a higher system power.

**Figure 9:**
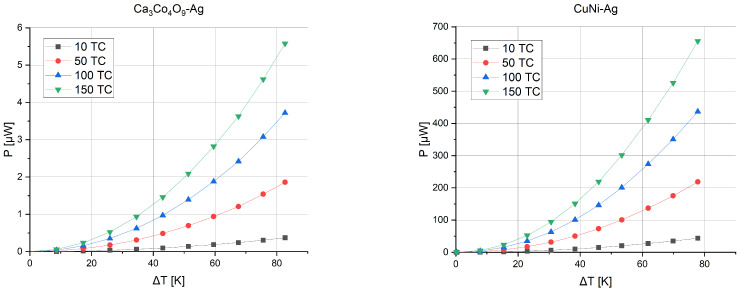
The generated power versus the temperature gradient and the number of thermocouples for resistance reduced by 50%.

For the test structure studied, the number of squares per thermocouple is about 118. When reducing this number to 59 (50%), the resistance of the structure will decrease proportionally, and thus more power will be generated (Figure 9).

By combining the two methods, it is possible to obtain the appropriate voltage and power required to power RFID tags in active or semi-passive mode. This is a promising application for ensuring an appropriate temperature gradient between the junctions and optimizing the thermoelectric microgenerator.

**Figure 10:**
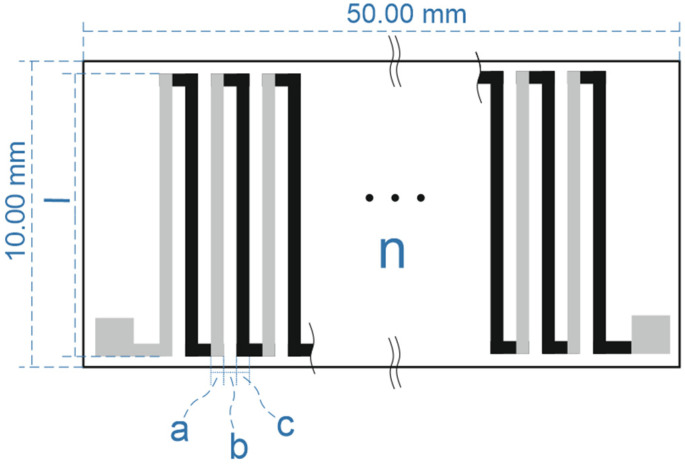
Schematic of a microgenerator on a substrate of size 10 × 50 mm^2^, where the following holds: a—width of the silver path; b—gap between paths; c—width of the constantan path; l—length of the single path; n—number of thermocouples.

On the basis of simulations, it was concluded that, given the relatively high power of the CuNi-Ag structure, it is appropriate to carefully consider the application of this microgenerator. In further analysis, a 10 × 50 mm^2^ substrate was adopted and simulations were carried out for four different thermocouple sizes. In the calculations sheet resistance, R□ = 0.060 [Ω/□] was taken for CuNi- and R□ = 0.012 [Ω/□] for Ag-based thick films. During the calculations, the sizes of the paths made of constantan and silver were modified. When determining the test cases, the possibilities associated with thick-film technology were taken into account. A schematic of the simulated structure is shown in Figure 10.

The four simulated structures are shown in Table 3, while the corresponding resistances are shown in Table 4. Structure 4 was specifically designed so that the resistances of the two paths were similar.

**Figure 11:**
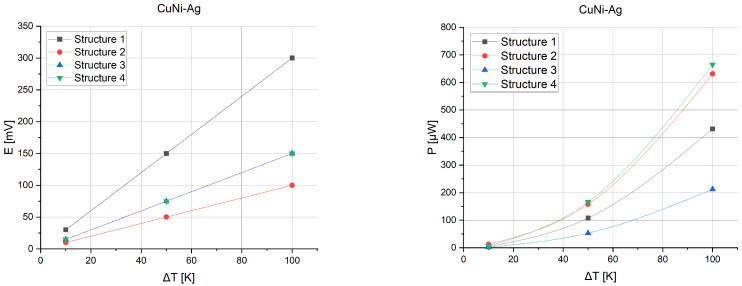
Voltage and power for four simulated structures at three gradient points of 10 K, 50 K, and 100 K.

On the basis of the specified parameters, the thermoelectric force and power of the designed microgenerators were calculated. Figure 11 presents the voltages and powers possible from the test microgenerators.

**Figure 12:**
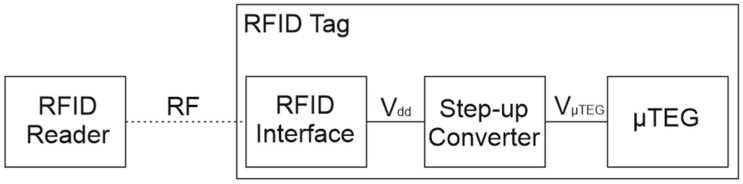
Schematic of μTEG’s connection to RFID.

The determined characteristics show that the best power performance is generated by the fourth structure, reaching about 170 μW at a 50 K gradient and about 670 μW at a 100 K gradient. On the basis of these results, it can be concluded that the microgenerator is suitable for use with RFID tags using a suitable step-up converter. During conversion with resistance matching, it must be assumed that the effective power will be lower according to relation 6. Figure 12 presents a schematic connection of the circuit to ensure correct operation. Table 5 shows the powers obtained with conversion for converters with 90% efficiency.

(6) $$P=\frac{E^{2}}{4\cdot R}$$

**Figure 13:**
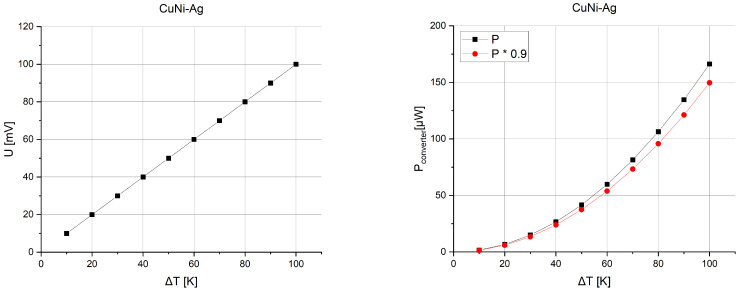
Voltage before conversion and effective power (100%, 90%) for the fourth structure.

According to the assumptions, it can be seen that with a voltage boost to 1.2 V, the microgenerator can provide adequate voltage and power for the RFID tag with resistance matching. It should be noted that a minimum power of 8 μW [14] has already been obtained with a gradient of less than 30 K (Figure 13).

By analyzing the presented structure, it is possible to show which gradient is necessary for the operation of the RFID tag per mm^2^ of the entire structure, starting from a gradient of 30 K since, as described above, this is considered the minimum required gradient (Table 6).

Based on the sources, the required energy per bit needed for specific communication protocols was estimated. According to the article [33], the approximate (averaged) energy was determined for the RTF, TTF, and 808.15.4 protocols. By approximating the results, the values presented in Table 7 were obtained.

**Figure 14:**
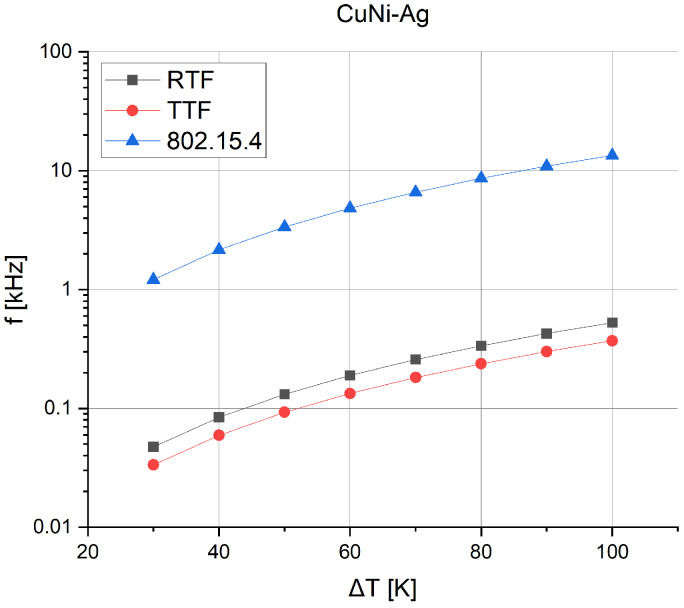
Bit transfer depends on the protocol and on the μTEG thermal gradient for the fourth structure.

Knowing the energy per bit, the number of bits that can be sent using a particular communication protocol was determined. Since the 808.15.4 protocol requires much less energy to transmit data, it allows a much higher number of bits to be sent. Figure 14 presents the frequency at which bits can be sent, depending on the thermal gradient.

With a one-second operating period assumed, the energy that the structure can generate depending on the gradient was determined. The results are summarized in Table 8.

## 7. Conclusions

This article presents an innovative approach to powering RFID tags through the use of planar μTEGs. Research confirms that these microgenerators can provide an efficient and sustainable power source for RFID tags, opening up new possibilities in the field of identification and monitoring technology.

The main conclusion of the research is that planar μTEGs can work well with RFID tags, enabling not only their continuous and autonomous operation but also eliminating the need for traditional power sources such as batteries. This results in increased reliability and durability of RFID-based identification systems.

According to the results, the Ca_3_Co_4_O_9_-based microgenerator only partially meets the criteria for working with active RFID tags. Despite the relatively high voltage generated by the Ca_3_Co_4_O_9_-Ag structure, its power is low, which is due to its high internal resistance. A structure providing adequate performance would have to operate with a very large temperature gradient or consist of more than 300–500 thermocouples, which can be very difficult technologically. With 150 thermocouples and a temperature gradient of 60 K, a voltage of 1.2 V is possible, which is a desirable result. However, the final power is only 1.5 μW, which is more than five times less than the minimum required value, which is 8 μW.

CuNi-based microgenerators show much higher output power; however, their voltage is below the minimum level required for an active RFID tag. For this reason, these structures were carefully studied for their promising power performance when planning their physical implementation. Four different types of structure were simulated on a 10 × 50 mm^2^ substrate. The best electrical performance was shown by a structure in which the width of the paths for silver was 0.2 mm and for constantan 1 mm, with a gap between them of 0.2 mm and a length of 9 mm. A maximum of 30 thermocouples fit on this structure, and all dimensions were adjusted according to the capabilities of thick film technology.

Simulations have shown that the CuNi-Ag structure is capable of working with RFID tags using a suitable boost converter that raises the voltage to 1.2 V. With a temperature gradient of 30 K and using a step-up converter with resistance similar to μTEG, 14.96 μW can be generated, suggesting the possibility of a practical application, assuming the minimum gradient is maintained.

Since different communication protocols require different amounts of energy for the transfer of a single data bit, an analysis of the frequency at which the tag can potentially send data bits depending on the power provided by the μTEG was conducted. According to the results, with a small thermal gradient (30 K), the microgenerator allows sending bits with suitable frequencies for RTF (~47 Hz), TTF (~33 Hz), and 802.15.4 (~1.2 kHz). As the thermal gradient increases, the effective power of the microgenerator also increases, resulting in a significant increase in frequency for the RTF (~0.53 kHz), TTF (~0.37 kHz), and 802.15.4 (~13.5 kHz) protocols at a gradient of 100 K.

From the results of this study, it can be concluded that the CuNi-Ag microgenerator can be effectively used to work with active or semi-passive RFID tags. As a result, it will make it possible to increase the communication distance and ensure continuous operation even in relatively low-temperature gradients.

On the basis of the research carried out, the practical implementation of the proposed solution of the thermoelectric microgenerator (μTEG) described above is planned. 

In conclusion, μTEGs combined with RFID tags present themselves as a promising solution that not only increases the energy efficiency of identification systems, but also fits the global drive to create environmentally friendly technologies. The introduction of these innovative solutions can contribute significantly to the development of RFID technology towards a sustainable future.

## Figures and Tables

**Table 1:** The structures studied.

Material 1	Application Process— Material 1	Isostatic Compression before Firing—Material 1	Firing— Material 1 [°C]	Material 2	Application Process— Material 2	Isostatic Compression before Firing—Material 2	Firing— Material 2 [°C]
Ca_3_Co_4_O_9_	Screen Printing	-	900	Ag	Screen Printing	-	500
Ca_3_Co_4_O_9_	Screen Printing	200 bar	900	Ag	Screen Printing	-	500
CuNi	Screen Printing	-	850	Ag	Screen Printing	-	500
CuNi	Screen Printing	200 bar	850	Ag	Screen Printing	-	500

**Table 2:** Resistance of a single thermocouple versus the temperature gradient (cold junctions at room temperature).

ΔT [K]	0	8	16	24	32	40	48	56	64	72	80
Ca_3_Co_4_O_9_-Ag-1b [kΩ]	7.079	7.032	6.983	6.930	6.878	6.818	6.763	6.704	6.643	6.578	6.486
CuNi-Ag-1b [Ω]	8.122	8.108	8.097	8.097	8.099	8.100	8.105	8.111	8.128	8.139	8.148

**Table 3:** Simulated structures with realistic dimensions.

Structure No.	a [mm]	b [mm]	c [mm]	l [mm]	n_max_ [-]
1	0.2	0.2	0.2	9	60
2	1	0.2	1	9	20
3	1	0.2	0.2	9	30
4	0.2	0.2	1	9	30

**Table 4:** Resistance of the simulated structures at the maximum number of thermocouples.

Structure No.	R_CuNi_ [Ω]	R_Ag_ [Ω]	R_CuNi_ + R_Ag_ [Ω]	Relationship
1	176.4	32.4	208.8	R_CuNi_ > R_Ag_
2	13.68	2.16	15.84	R_CuNi_ > R_Ag_
3	102.6	3.24	105.84	R_CuNi_ > R_Ag_
4	17.64	16.2	33.84	R_CuNi_ ≈ R_Ag_

**Table 5:** Comparison of voltage and power values before and after conversion for the fourth structure.

ΔT [K]	U—before Conversion [mV]	P_μTEG_—Effective with Load [μW]	U—after Conversion [mV]	P_converter_—after Conversion Where η = 90% [μW]
10	15	1.66	1200	1.50
20	30	6.65	1200	5.98
30	45	14.96	1200	13.46
40	60	26.60	1200	23.93
50	75	41.56	1200	37.40
60	90	59.84	1200	53.86
70	105	81.45	1200	73.30
80	120	106.38	1200	95.74
90	135	134.64	1200	121.18
100	150	166.22	1200	149.60

**Table 6:** Power distribution per mm^2^ of structure, for the fourth structure with dimensions of 10 × 50 mm^2^.

ΔT [K]	30	40	50	60	70	80	90	100
P/A [μW/mm^2^]	0.0299	0.0532	0.0831	0.1197	0.1629	0.2128	0.2693	0.3324

**Table 7:** Energy estimated per bit relative to the protocol.

Protocol	RTF	TTF	808.15.4
E_b_ [nJ/bit]	315.91	448.16	12.33

**Table 8:** Energy distribution per mm^2^ of the structure, for the fourth structure with dimensions of 10 × 50 mm^2^.

ΔT [K]	30	40	50	60	70	80	90	100
E/A [nJ/mm^2^]	29.920	53.191	83.112	119.681	162.899	212.766	269.282	332.447

## Data Availability

Data are contained within the article.
